# Transarterial RAdioembolization versus ChemoEmbolization for the treatment of hepatocellular carcinoma (TRACE): study protocol for a randomized controlled trial

**DOI:** 10.1186/1745-6215-13-144

**Published:** 2012-08-23

**Authors:** Beatrijs A Seinstra, Luc Defreyne, Bieke Lambert, MarnixGEHH Lam, Helena M Verkooijen, Karel J van Erpecum, Bart van Hoek, Arian R van Erkel, Minneke J Coenraad, Imad Al Younis, Hans van Vlierberghe, MauriceAAJ van den Bosch

**Affiliations:** 1 Department of Radiology, University Medical Center Utrecht, Room E.01.132, Heidelberglaan 100, 3584, CX, Utrecht, The Netherlands; 2 Department of Gastroenterology and Hepatology, University Medical Center Utrecht, Utrecht, The Netherlands; 3 Department of Nuclear Medicine, University Medical Center Utrecht, Utrecht, The Netherlands; 4 Department of Vascular and Interventional Radiology, Ghent University Hospital, Ghent, Belgium; 5 Department of Nuclear Medicine, Ghent University Hospital, Ghent, Belgium; 6 Department of Gastroenterology and Hepatology, Ghent University Hospital, Ghent, Belgium; 7 Department of Radiology, Leiden University Medical Center, Leiden, The Netherlands; 8 Department of Gastroenterology and Hepatology, Leiden University Medical Center, Leiden, The Netherlands; 9 Department of Nuclear Medicine, Leiden University Medical Center, Leiden, The Netherlands

## Abstract

**Background:**

Hepatocellular carcinoma is a primary malignant tumor of the liver that accounts for an important health problem worldwide. Only 10 to 15% of hepatocellular carcinoma patients are suitable candidates for treatment with curative intent, such as hepatic resection and liver transplantation. A majority of patients have locally advanced, liver restricted disease (Barcelona Clinic Liver Cancer (BCLC) staging system intermediate stage). Transarterial loco regional treatment modalities offer palliative treatment options for these patients; transarterial chemoembolization (TACE) is the current standard treatment. During TACE, a catheter is advanced into the branches of the hepatic artery supplying the tumor, and a combination of embolic material and chemotherapeutics is delivered through the catheter directly into the tumor. Yttrium-90 radioembolization (^90^Y-RE) involves the transarterial administration of minimally embolic microspheres loaded with Yttrium-90, a β-emitting isotope, delivering selective internal radiation to the tumor. ^90^Y-RE is increasingly used in clinical practice for treatment of intermediate stage hepatocellular carcinoma, but its efficacy has never been prospectively compared to that of the standard treatment (TACE). In this study, we describe the protocol of a multicenter randomized controlled trial aimed at comparing the effectiveness of TACE and ^90^Y-RE for treatment of patients with unresectable (BCLC intermediate stage) hepatocellular carcinoma.

**Methods/design:**

In this pragmatic randomized controlled trial, 140 patients with unresectable (BCLC intermediate stage) hepatocellular carcinoma, with Eastern Cooperative Oncology Group performance status 0 to 1 and Child-Pugh A to B will be randomly assigned to either ^90^Y-RE or TACE with drug eluting beads. Patients assigned to ^90^Y-RE will first receive a diagnostic angiography, followed by the actual transarterial treatment, which can be divided into two sessions in case of bilobar disease. Patients assigned to TACE will receive a maximum of three consecutive transarterial treatment sessions. Patients will undergo structural follow-up for a timeframe of two years post treatment. Post procedural magnetic resonance imaging (MRI) will be performed at one and three months post trial entry and at three-monthly intervals thereafter for two years to assess tumor response. Primary outcome will be time to progression. Secondary outcomes will be overall survival, tumor response according to the modified RECIST criteria, toxicities/adverse events, treatment related effect on total liver function, quality of life, treatment-related costs and cost-effectiveness.

**Trial registration:**

NCT01381211

## Background

Hepatocellular carcinoma (HCC), a primary malignant tumor of the liver, is the sixth most common cancer worldwide with an incidence of 626,000 new patients a year, and the third most common cause of cancer-related death [1]. HCC is a heterogeneous disease in terms of etiology and clinical behavior. It usually develops in the setting of chronic liver disease, mostly related to infection with hepatitis B or C, excessive alcohol intake and, today, also more as a consequence of non-alcoholic steatohepatitis (NASH) [2]. Cure can only be achieved by hepatic resection, liver transplantation and in certain cases by radiofrequency ablation (RFA) [3]. Although there is no universally accepted HCC staging system, many have adopted the Barcelona Clinic Liver Cancer (BCLC) staging classification, which links the stage of the disease to a specific treatment strategy [4,5].

Only 10% to 15% of all patients with HCC are eligible for treatment with curative intent, and local ablative treatment is limited by strict criteria on the stage of the disease (that is, disease confined to the liver and limited number of lesions with relatively small dimensions (maximum of three lesions <3 cm in size) according to the BCLC staging classification). However, the majority of patients present at more advanced stages and are not eligible for such treatment.

For patients with locally advanced, liver restricted disease (BCLC intermediate stage), transarterial loco regional treatment modalities offer palliative treatment options. These therapies exploit the dual blood supply to the liver. HCC derives its blood supply almost entirely from the hepatic artery, while liver parenchyma derives >75% of its blood supply from the portal vein [6]. This anatomical fact provides the basis for the development of intra-arterial therapies for the treatment of HCC, with the potential of selectively inducing tumor necrosis while sparing surrounding liver parenchyma. Antitumoral agents, such as cytotoxic drugs or radionuclides, can be delivered at the site of the tumor, as they lodge in the peritumoral vascular bed after intra-arterial injection.

This randomized controlled trial is designed to compare two transarterial loco regional therapies applied for the treatment of patients with intermediate stage HCC: transarterial chemoembolization (TACE), the current standard treatment, and Yttrium^-^90 radioembolization (^90^Y-RE), a newer treatment modality.

### Transarterial chemoembolization

Transarterial chemoembolization (TACE) is a procedure in which a catheter is advanced into the branches of the hepatic artery supplying the tumor, and a combination of embolic material and chemotherapeutics is delivered through the catheter directly into the tumor. This way arterial inflow to the tumor is reduced, resulting in ischemic tumor necrosis, and washout of the chemotherapeutic agent is diminished, thereby prolonging contact time between cancer cells and the drug [7].

According to the BCLC staging classification and treatment schedule, TACE can be considered the current standard treatment for patients with intermediate stage HCC with compensated liver disease, with a reported median survival of around 17 months [8-10].

Two randomized, controlled trials [11,12] and two systematic reviews [13,14] demonstrated a survival benefit of TACE in this selected patient group. A more recent Cochrane review and meta-analysis concluded that there is no firm evidence to support or refute TACE in patients with unresectable HCC [15]. However, this review is controversial since some studies included in the meta-analysis included patients that are no longer consistent with the more stringent selection criteria currently applied for TACE (BCLC intermediate stage B with compensated liver disease) and there are discrepancies in treatment application between the studies. The lack of standardization accompanied with the application of conventional TACE (mostly performed with a mixture of chemotherapeutics, lipiodol and an occluding agent) is no longer an issue with the arrival of drug-eluting beads (DEBs). DEBs act as both an occluding agent as well as a drug-loaded carrier, achieving local ischemia and cytotoxic death of the tumor with one device, enabling standardization [16]. Several clinical trials reported DEBs to be effective for treatment of intermediate stage HCC [16-20], with objective response (complete plus partial response) rates ranging from 60% to 85.5%, which is substantially higher compared to the mean objective response of 35% (range 16% to 61%) stated in a meta-analysis of RCTs for conventional TACE [14]. The complication rates are considered acceptable, although post-embolization syndrome is observed in 37% to 100% of the treated patients. This is a condition in which the patient experiences abdominal pain, fever, ileus and nausea, self-limiting hours to days after the procedure, probably due to damage of hepatocytes [21]. Most importantly, these trials report no systemic toxicity despite the high doses of doxorubicin loaded on the DEBs [16-20]. The PRECISION V trial compared conventional TACE with TACE-DEB in a large, randomized study [22]. The TACE-DEB group showed higher rates of complete response, objective response and disease control when compared with conventional TACE at six months (27% vs. 22%, 52% vs. 44%, 63% vs. 52%); however, the difference was not statistically significant. Subgroup analysis showed that TACE-DEB results in a higher tumor control rate in patients with Child-Pugh B cirrhosis, bilobar disease or reduced performance score compared to conventional TACE. This was achieved without an increased risk of adverse events [15].

### Yttrium-90 radioembolization

Yttrium-90 radioembolization (^90^Y-RE) is a relatively recently developed technique which involves the transarterial administration of minimally embolic microspheres loaded with Yttrium-90, a β-emitting isotope, delivering selective internal radiation to the tumor. This brachytherapy device is approved by the Food and Drug Administration for HCC with and without portal vein thrombosis (PVT). Several prospective and retrospective studies demonstrated the safety and efficacy of 90Y-RE treatment for unresectable HCC, all documented in a recent review [23]. An earlier structured meta-analysis describes a response rate of 78% (glass microspheres) and 89% (resin microspheres) [24]. The largest prospective study to date included 291 HCC patients that were treated with ^90^Y-RE [25]. In intermediate stage patients (BCLC stage B) median time to progression was 13.3 months (Child-Pugh A, 13.3 months and Child-Pugh B, 17.4 months) and median survival was 17.2 months (Child-Pugh A, 17.3 months and Child-Pugh B, 13.5 months).

### Study rationale

Although ^90^Y-RE is increasingly used in clinical practice, there is no high quality clinical evidence to justify this. Lewandowsky *et al. *[10] retrospectively analyzed HCC patients with disease beyond the Milan criteria for liver transplantation and concluded that radioembolization outperforms TACE for down-staging HCC to within transplant criteria. The results of a single-center study carried out by Kooby *et al. *[26], in which patients treated with chemoembolization or radioembolization were retrospectively compared, suggests that both treatment modalities have similar effectiveness and safety profiles in patients with unresectable HCC. Carr *et al. *[27] carried out a similar retrospective analysis and concluded that chemoembolization or radioembolization appeared to be equivalent regional therapies for patients with unresectable, nonmetastatic HCC. Recently Salem *et al. *[28] retrospectively compared 122 HCC patients who received chemoembolization with 123 patients who received radioembolization and concluded both patient groups had similar survival times. Radioembolization resulted in longer time-to-progression and less toxicity than chemoembolization.

To date, no prospective studies have been performed comparing both treatment modalities in a randomized setting. This randomized controlled trial is designed to prospectively compare TACE and ^90^Y-RE for treatment of patients with unresectable (BCLC intermediate stage) HCC.

## Methods

### Study participants

Patients with intermediate stage hepatocellular carcinoma (BCLC class B; locally advanced, liver restricted disease, not eligible for curative treatment) with Eastern Cooperative Oncology Group (ECOG) performance status 0 to 1 and Child-Pugh A to B and written informed consent will be eligible for inclusion. The HCC diagnosis will be confirmed by typical appearance on imaging (four-phase multidetector CT scan or dynamic contrast enhanced magnetic resonance imaging (MRI)), that is, hyper vascular enhancing lesion in the arterial phase and contrast washout in the portal venous or delayed phase, or by cytohistological tissue sampling by biopsy in case of inconclusive imaging findings [29]. Exclusion criteria to be applied are lack of informed consent, extrahepatic disease, abnormal organ or bone marrow function (as determined by bilirubin >45 μmol/l (or 2.6 mg/dl), serum albumin <28 g/l, AST/ALT >5x institutional upper limit of normal (ULN), creatinine >1.5x institutional ULN, hemoglobulin <6.0 mmol/l, absolute neutrophil count <1.5 x 10^9^/l, platelet count <60 x 10^9^/l), compromised biliary system, hypersensivity to doxorubicin, pregnancy or breast feeding, >50% of liver involvement, main portal vein (right, left or common trunk) thrombosis, unmanageable intolerance for contrast medium, life expectancy less than three months or otherwise impossible follow-up. Previous local therapy for HCC will not be a contraindication if at least one measurable target lesion is present, which has not been treated previously.

### Study design

This is a pragmatic, multicenter, randomized controlled trial. Patients will be recruited after referral by their hepatologist or another treating physician. Treatment allocation will be performed by minimization, which is a valid alternative to standard randomization to ensure balance between groups for several prognostic factors [30]. The following prognosis-related patient characteristics will be taken into account: Child-Pugh stage (A/B), ECOG performance status (0/1), prior curative (resection or percutaneous ablation) treatment (yes/no) and bilobar disease (yes/no). The minimization procedure will be stratified by treatment center, with the aid of validated software, whereby a random component (10%) is to be introduced.

### Assessment before treatment

Pre-procedural MRI will be performed within four weeks prior to the date of minimization to define HCC lesions by number, volume and location.

### Intervention

#### TACE

TACE will be performed with drug-eluting beads, that is, polyvinyl alcohol-based microspheres (DC Bead®, Biocompatibles, Farnham, UK), 100 to 300 μm in diameter, loaded with the chemotherapeutic agent doxorubicin. Patients will receive a maximum dose of 150 mg doxorubicin per single treatment session. No dose adjustment will be made for bilirubin concentration or body surface area. Dose determination is based on previous dose escalation studies, in which DEBs loaded with doxorubicin up to 150 mg were shown to be safe and effective [19,20], and recommendations from the literature [17,22]. After mixing with nonionic contrast medium, the DEBs will be slowly injected under fluoroscopic visualization while the contrast flow rate is observed; the embolization endpoint will be reached when all the DEBs are administered (maximum dose is reached), or earlier when sluggish flow is seen (to avoid reflux and non-target embolization). Total administered dose will be recorded.

Injection of DEBs will be performed as selectively as possible after Cone-Beam CT confirmed perfusion of the target lesions. If selective administration is not possible, a lobar treatment will be performed. The TACE procedure will be repeated after two months. A third TACE at four months after initial treatment will only be performed in the case of residual enhancement on the three months follow-up contrast-enhanced MRI scan of the liver. The moment of the first treatment will be defined as trial entry.

#### ^90^Y-RE

^90^Y-RE will be performed using glass Yttrium-90 microspheres (TheraSphere®, MDS Nordion, Ottawa, Ontario, Canada). The target dose to the liver will be 100 to 120 Gy. The procedure will be carried out over two separate sessions: a work-up session and a treatment session. Meticulous work-up is very important because it determines the overall safety of the treatment. First, a selective visceral catheterization will be performed in order to obtain an angiographic map of the patients’ vascular anatomy. Branches feeding gastro-intestinal organs arising from the hepatic arteries must be identified. Radioactive microspheres, administered into the hepatic artery, should be prevented from ending up in extrahepatic organs via this route. The interventional radiologist will actively look for the presence of the gastroduodenal artery, right gastric artery, cystic artery and pancreaticoduodenal branches. If required, these arteries will be embolized using coils, taking the planned catheter position for injecting the microspheres into account. Additionally, 150 MBq Technetium-99 m-labeled macro aggregated albumin (^99m^Tc-MAA) will be injected. ^99m^Tc-MAA is used as a surrogate in order to predict the distribution pattern of ^90^Y-microspheres [31]. The distribution of ^99m^Tc-MAA will be visualized by whole body planar imaging and single-photon emission computed tomography (SPECT-CT), including low dose computer tomography of the abdomen. Accordingly, lung shunt fraction can be calculated and deposition of ^99m^Tc-MAA in the abdominal organs, such as the stomach, duodenum and pancreas, can indicate patent extrahepatic vessels distal to the injection site. In case a lung dose exceeding 30 Gy (610 MBq) in a single treatment (or 50 Gy cumulatively in case of repeated treatment) is predicted, an activity reduction will be prescribed [32].

In case a patient has an unfavorable ^99m^Tc scintigraphy, the ^99m^Tc-MAA workup procedure will be repeated, if feasible, to detect the cause of the extrahepatic deposition (for example, previously undetected patent extrahepatic vessels arising from the hepatic artery) and a solution will be searched for (for example, more selective placement of the catheter during injection). In the unlikely event no solution can be found and ^90^Y-RE cannot be performed, the patient will be treated according to best medical practice and will still enter the intention-to-treat analysis in the ^90^Y-RE arm.

When the ^99m^Tc-MAA scintigraphy has a favorable outcome, patients will be readmitted to the hospital for the treatment session within two weeks. The volume of liver lobe to be treated and corresponding liver mass will be determined using CT or MRI. The activity is chosen to deliver an absorbed dose of about 120 Gy to the treatment zone in patients with no manifest risk factors for subsequent liver decomposition. If a patient presents at inclusion with elevated bilirubin or Child-Pugh B status, the activity will be reduced in order to reach an absorbed dose of 80 to −100 Gy in the target volume, but in the case of selective treatment on a segmental level a dose up to 150 Gy may be administered. The radioactivity required to deliver the desired dose to the liver will be calculated using the following formula:

$$\text{Activity Required}\;\left(\text{GBq}\right)=\frac{\left[\text{Desired}\;\text{Dose}\left(Gy\right)\right]\;\left[\text{Liver}\;\text{Mass}\left(kg\right)\right]}{50}$$

The actual liver dose (Gy) delivered to the liver after injection can be calculated using the following formula:

$$\text{Dose}\;\left(Gy\right)=\frac{50\;\left[\text{Injected}\;\text{Activity}\left(GBq\right)\right]\;\left[1-F\right]}{\text{Liver}\;\text{Mass}\left(kg\right)}$$

where F is the fraction of injected radioactivity localizing in the lungs, as measured by ^99m^Tc-MAA scintigraphy.

The hepatic artery will be catheterized and the ^90^Y-microspheres will be administered from the exact same microcatheter position as where the ^99m^Tc-MAA was administered. Injection of ^99m^Tc-MAA and ^90^Y therapy will be performed as selectively as possible. In case of bilobar disease, the right and left lobe will be treated in two separate sessions, with a generally accepted interval of 30 to 45 days. The moment of the first treatment will be regarded trial entry.

### Follow-up

Patients will undergo structural follow-up for a timeframe of two years post treatment. Each patient will be seen by the treating physician during regular visits to the outpatient clinic, during which laboratory examination will also be performed (complete blood count, electrolytes, kidney function tests, liver function tests, albumin, Prothrombin time/INR, alpha-fetoprotein). Post-procedural, contrast-enhanced MRI will be performed one month after trial entry (the date of the first treatment T0), three months after trial entry and at three monthly intervals thereafter for two years to assess tumor response. MRI will be performed on 1.5-T scanners using a spoiled gradient-echo T1-weighted sequence and fast spin-echo T2-weighted sequence with fat suppression. A dynamic multiphase, contrast-enhanced, spoiled gradient-echo T1-weighted sequence with arterial, portal, equilibrium and delayed phase will be performed. In patients in which MRI is contra-indicated or who are not capable of breath holding, contrast enhanced CT will be carried out. Adverse events and toxicities will be recorded according to the National Cancer Institute Common Terminology Criteria for Adverse Events (CTCAE version 4.0). These criteria define adverse events on a scale from 1 to 5, corresponding to severity (grade 1: mild, grade 2: moderate, grade 3: severe, grade 4: life-threatening or disabling, grade 5: resulting in death). Toxicity will only be recorded if the grade increases from baseline. Adverse events and toxicities will be monitored for six months following the last treatment procedure. Patients will be asked to fill out questionnaires for quality of life assessment before treatment and at 1, 3, 6 and 12 months after first treatment. Quality of life will be measured with the EuroQol Group 5-Dimension Self-Report Questionnaire (EQ-5D), the cancer specific EORTC QLQ-C30 and its HCC specific supplement EORTC QLQ-HCC18 and an abbreviated version of the Short Form – Health and Labor Questionnaire (SF-HLQ).

### Objective

The objective of this study is to compare the efficacy and safety of TACE versus ^90^Y-RE in patients with intermediate stage HCC.

### Outcomes

#### Primary outcome

The primary outcome will be Time to Progression (TTP), defined as the time elapsed since the start of treatment until the determination of progressive disease. Progressive disease will be determined by image evaluation according to the modified RECIST (mRECIST) response evaluation criteria [33]. The mRECIST overall response assessment includes the evaluation of target lesions response, non-target lesions response and the occurrence of new lesions.

The baseline for calculation of TTP will be the date of minimization. Imaging data will be assessed by independent radiologists. In case progressive disease is determined during follow-up, patients have reached the study endpoint and will be treated according to best medical practice. Patients who switch to other treatments are registered as such and overall survival will be monitored.

#### Secondary outcomes

1: Time to Local Progression (TLP). ‘Target liver volume’ will be determined with the use of cone-beam CT during the interventional procedure, by implementing the perfusion area of the targeted vessel in relationship to the anatomic localization of the liver lesion(s). TLP will be defined as the time elapsed since the start of treatment (baseline is the date of minimization) until *local* tumor progression, ‘local’ indicating a constriction to the target liver volume as defined by cone-beam CT. Local tumor progression will be based on the mRECIST criteria and defined as: An increase of at least 20% in the sum of the diameters of viable (enhancing) target lesions (whereas target lesions for TLP evaluation are only considered within the ‘target liver volume’), taking as a reference the smallest sum of the diameters of viable (enhancing) target lesions recorded since treatment started, and/or unequivocal progression of existing no target lesions within this perfusion area and/or the appearance of one or more new (enhancing) lesions within this perfusion area; 2: Overall survival; 3: Overall response to therapy according to mRECIST; 4: Toxicities and adverse events; 5: Quality of life; 6: Treatment-related costs, in terms of cost of therapy and number of hospitalization days, in these patients. Cost-effectiveness of ^90^Y-RE versus TACE will be assessed.

#### Sample size calculation

A total of 140 patients will be included. Sample size calculation was performed using PASS (Power Analysis and Sample Size software, NCSS, Kaysville, Utah, USA), using the log rank test. Assuming a follow-up time of 24 months and a median time to progression of 13.3 months after ^90^Y-RE, based on the best stratified data for intermediate stage HCC patients available in the literature [25], a clinically relevant effect size of 20% difference in time to progression (2.7 months) with a power of 90% and a two-sided Alpha Risk of 5%, 70 patients in each treatment arm will be required. Taking a hypothetical loss to follow-up of 2% into account in both treatment arms, based on earlier experience with this patient group, still gives a power of 80% if 70 patients per treatment arm are included (Alpha Risk remains 5%).

#### Null hypothesis

There is no difference in TTP in patients with intermediate stage HCC treated with ^90^Y-RE or TACE-DEB.

The alternative hypothesis is two-sided (^90^Y-RE could have a shorter or a longer TTP).

#### Statistical analyses

The primary outcome of the study will be time to progression. Kaplan-Meier analysis will be performed to compare time to progression between treatment arms and difference in TTP will be tested by using the log rank test. The Hazard Ratio (and 95% confidence interval) will be calculated by univariate analyses. A *P-*value of less than .05 will be considered to be statistically significant. Analyses will be performed according to the intention to treat principle. In addition, per protocol analysis will be performed, including only those patients who completed the treatment protocol originally allocated. In terms of secondary outcomes, we will analyze time to local progression and overall survival by means of Kaplan Meier analysis, and test differences between the two arms with log rank test. Differences in proportions of patients reporting toxic/adverse events or treatment related complications will be analyzed by means of a chi square test. Differences in quality of life (as measured by standard questionnaires) over time between the two arms will be analyzed by means of Linear Mixed Model analysis. Statistical analysis of results will be performed with the aid of SPSS software (SPSS Inc., Chicago, Illinois, USA).

## Trial status

The trial is currently including patients. The first 10 patients have been minimized and treated according to the allocated treatment arm. Until the present all patients adhered to the trial protocol. First results are pending.

## Ethical approval

The study protocol has been approved by the ethical boards of the participating hospitals.

## Abbreviations

^90^Y-RE: Yttrium-90 radioembolization; ^99m^Tc-MAA: Technetium-99 m-labeled macro aggregated albumin; ALT: alanine aminotransferase; AST: aspartate aminotrasferase; BCLC: Barcelona Clinic Liver Cancer; CT: computed tomography; CTCAE: National Cancer Institute Common Terminology Criteria for Adverse Events; DEB: drug eluting bead; ECOG: Eastern Cooperative Oncology Group; HCC: hepatocellular carcinoma; MRI: Magnetic Resonance Imaging; RECIST: Response Evaluation Criteria in Solid Tumors; SPECT: single-photon emission computed tomography; TACE: transarterial chemoembolization; TTP: time to progression; ULN: upper limit of normal.

## Competing interests

The authors declare that they have no competing interests.

## Authors’ contributions

BAS drafted the manuscript and contributed to the design of the study. LD, BL and MGEHL contributed to the design of the study and helped to draft the manuscript. HMV contributed to the design of the study, helped out with the statistical analysis and helped to draft the manuscript. KJE, BvH, ARE, MJC, IAY and HV contributed to the design of the study. MAAJB conceived of the study, contributed to its design and helped to draft the manuscript. All authors read and approved the final manuscript.
